# Association of ultrasound-guided fascia iliaca compartment block with sedation and postoperative nausea and vomiting in elderly patients undergoing hip arthroplasty: A retrospective propensity score–matched cohort study

**DOI:** 10.1097/MD.0000000000049855

**Published:** 2026-07-31

**Authors:** Qingtang Yin, Daobing Chen, Chunmiao Gu, Beibei Zhang

**Affiliations:** a Anesthesiology Department, Haimen District People’s Hospital, Nantong City, Jiangsu Province, China.

**Keywords:** elderly, hip arthroplasty, postoperative nausea and vomiting, regional nerve block, sedation

## Abstract

This study aimed to compare the association of ultrasound-guided fascia iliaca compartment block (FICB) with sedation versus conventional general anesthesia (GA) with postoperative nausea and vomiting (PONV) in elderly patients undergoing hip arthroplasty. This retrospective propensity score–matched cohort study included patients aged ≥ 60 years who underwent elective unilateral hip arthroplasty between March 2022 and March 2024. Patients were classified according to the anesthesia technique received: ultrasound-guided FICB with dexmedetomidine-based sedation or conventional endotracheal GA. Propensity score matching was performed to balance baseline characteristics. The primary outcome was the incidence of PONV within 24 hours postoperatively. Secondary outcomes included PONV requiring pharmacological intervention, early PONV within 0 to 6 hours, opioid consumption, time to first analgesic request, post-anesthesia care unit (PACU) stay, time to ambulation, and anesthesia-related adverse events. After matching, 100 patients were included, with 50 patients in each group. The FICB-sedation group had a lower 24 hours PONV incidence than the GA group (16.0% vs 42.0%, *P* = .004), corresponding to an absolute risk reduction of 26.0% and a number needed to treat of approximately 4. PONV requiring pharmacological intervention was also lower in the FICB-sedation group (14.0% vs 36.0%, *P* = .012), as was early PONV within 0 to 6 hours postoperatively (10.0% vs 30.0%, *P* = .012). The FICB-sedation group showed reduced intraoperative and postoperative opioid consumption, delayed first analgesic request, shorter PACU stay, and earlier ambulation. In multivariable logistic regression, FICB with sedation was independently associated with a lower risk of PONV, whereas higher postoperative opioid consumption was associated with increased PONV risk. The incidence of intraoperative hypotension was lower in the FICB-sedation group. In elderly patients undergoing hip arthroplasty, ultrasound-guided FICB with sedation was associated with a lower incidence and severity of PONV, reduced opioid consumption, faster early recovery, and improved intraoperative hemodynamic stability compared with conventional GA. Given the retrospective single-center design, these findings should be interpreted as associative and require confirmation in prospective randomized studies.

## 1. Introduction

Postoperative nausea and vomiting (PONV) is one of the most common adverse effects following general anesthesia (GA). Its incidence can be as high as 30 to 50% in elderly patients undergoing major orthopedic surgeries, particularly hip arthroplasty.^[1,2]^ PONV not only causes significant patient discomfort, prolongs post-anesthesia care unit (PACU) stay, and increases the risk of unplanned hospitalization, but in the elderly population, it may also trigger aspiration, exacerbate cardiovascular strain, and severely impair the postoperative recovery experience and early functional exercise.^[3,4]^

The extensive use of opioids is considered the primary pharmacological factor contributing to PONV.^[5]^ Traditional GA regimens rely heavily on potent opioids (such as sufentanil, remifentanil) for intraoperative analgesia, forming a key basis for postoperative PONV. Consequently, opioid-sparing multimodal analgesia strategies have become a crucial component of modern enhanced recovery after surgery (ERAS) protocols.^[6,7]^

In recent years, the widespread adoption of ultrasound-guided regional nerve block techniques has revolutionized analgesia for orthopedic surgeries. Among these, the fascia iliaca compartment block (FICB), due to its relative simplicity and high safety profile in effectively covering sensory nerves of the hip and anterolateral thigh, has become a key perioperative analgesic technique for hip surgeries.^[8]^ Utilizing this block as the primary analgesic method, combined with light sedation (e.g., dexmedetomidine) to complete the surgery, theoretically offers a highly promising solution. It can significantly reduce intraoperative and postoperative opioid requirements from the source, thereby potentially lowering the incidence of PONV.

However, there is still relatively limited high-quality retrospective evidence directly comparing the impact of this “block-sedation” approach versus conventional GA on PONV in elderly patients undergoing hip arthroplasty, particularly studies that employ propensity score matching to control for confounding factors. Recent evidence supports the potential role of regional or neuraxial anesthesia in reducing opioid exposure and selected patient-centered adverse outcomes after hip surgery, although findings vary according to surgical population, regional technique, and perioperative care pathway. A meta-analysis^[9]^ comparing spinal and GA in total hip arthroplasty reported a lower occurrence of postoperative nausea with spinal anesthesia, while more recent evidence on FICB has consistently suggested opioid-sparing effects but less consistent effects on PONV itself. This inconsistency indicates that PONV may be influenced not only by the regional block but also by sedation strategy, opioid exposure, antiemetic practice, and ERAS-related care. Therefore, further real-world evidence focusing specifically on PONV after block-sedation techniques in elderly hip arthroplasty patients remains clinically relevant.

Therefore, this study aims to systematically compare the effects of ultrasound-guided FICB combined with sedation versus conventional GA on the incidence, severity, and opioid consumption related to PONV in elderly hip arthroplasty patients. Using a retrospective cohort design with propensity score matching, the study seeks to provide evidence-based insights for optimizing clinical anesthesia protocols and improving patient outcomes.

## 2. Methods

### 2.1. Study design

**Figure 1.**
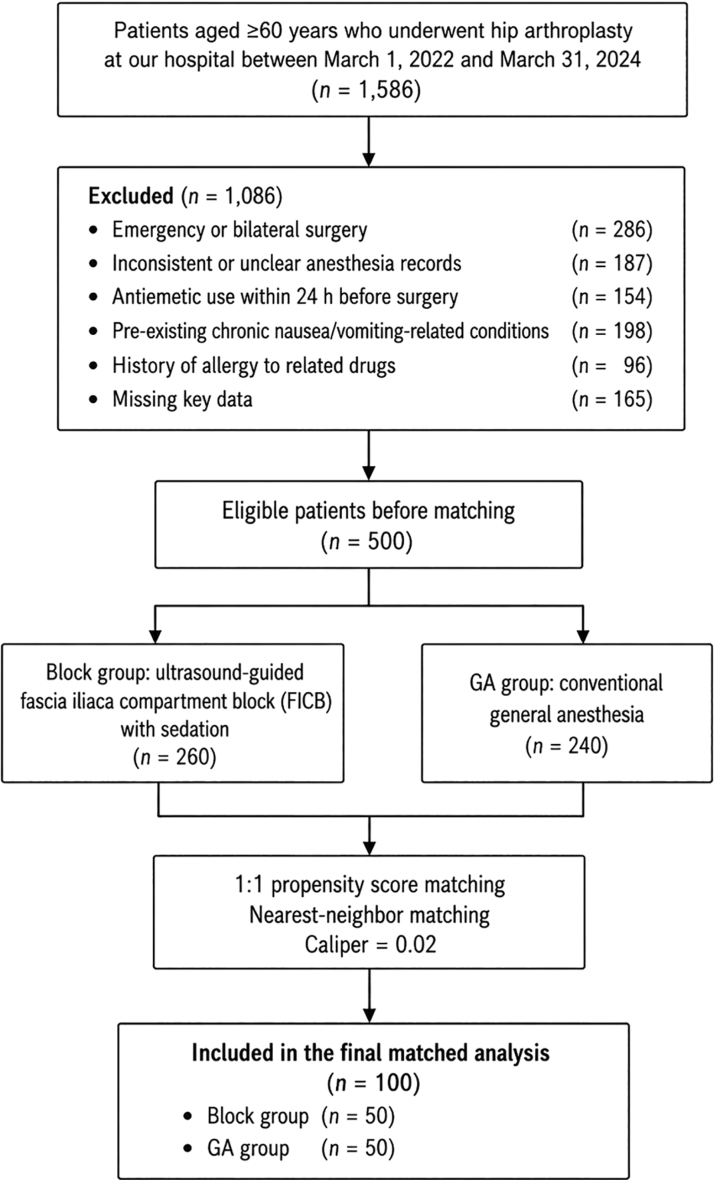
Flow diagram of patient selection and propensity score matching.

This study was approved by the Ethics Committee of Haimen District People’s Hospital, Nantong City. This study is a retrospective cohort study. We systematically reviewed the clinical data of elderly patients (aged ≥ 60 years) who underwent primary, unilateral hip arthroplasty at our hospital between March 1, 2022, and March 31, 2024. Based on the actual anesthesia protocol received, patients were divided into 2 groups: the regional nerve block combined with sedation group (hereinafter referred to as the “Block Group”) and the conventional GA group (hereinafter referred to as the “GA Group”). To control for potential confounding bias inherent in retrospective studies and to ensure comparability of baseline characteristics between the groups, we employed propensity score matching for subject screening and pairing. After matching, a total of 50 patients in each group with balanced baselines were included in the final analysis (Fig. 1). The study protocol was approved by the Ethics Committee of our hospital, and the requirement for informed consent was waived.

### 2.2. Group definition

#### 2.2.1. General anesthesia group (n = 50)

Upon entering the operating room, patients underwent standard vital sign monitoring (noninvasive blood pressure, electrocardiogram, pulse oximetry), and intravenous access was established. Anesthesia induction was performed using etomidate (0.2–0.3 mg/kg), sufentanil (0.3–0.5 μg/kg), and vecuronium (0.1 mg/kg). Orotracheal intubation was followed by mechanical ventilation. Intraoperative anesthesia maintenance was achieved with continuous intravenous infusion of propofol (4–12 mg/kg/h), supplemented by additional sufentanil as needed, based on surgical stimulation and vital sign changes, to maintain analgesia.

#### 2.2.2. Block group (n = 50)

Patient monitoring upon entering the operating room was identical to that in the General Anesthesia Group. Prior to anesthesia induction, while under conscious sedation, an ultrasound-guided unilateral FICB was performed first. The success of FICB was assessed 15 to 20 minutes after local anesthetic injection and before surgical incision. Block efficacy was evaluated using loss or reduction of cold or pinprick sensation over the femoral and lateral femoral cutaneous nerve territories, together with patient-reported pain relief during passive hip movement when feasible. Surgery was initiated only after clinically adequate analgesia was confirmed. Cases with absent, failed, or insufficiently documented block effect were excluded as unclear or inconsistent anesthesia records. A single injection of 30 mL of 0.25% ropivacaine was administered. Surgery commenced after confirmation of the analgesic effect from the block. Intraoperative sedation was maintained with intravenous infusion of dexmedetomidine (loading dose 0.5–1.0 μg/kg, followed by continuous infusion at 0.2–0.7 μg/kg/h). Intraoperative sedation was maintained with dexmedetomidine infusion, with low-dose propofol administered when necessary. Sedation depth was assessed and documented by the attending anesthesiologist using the Ramsay sedation scale, targeting a score of 3 to 4 throughout surgery. Hemodynamic variables, respiratory rate, pulse oximetry, and level of consciousness were continuously monitored. Bispectral index monitoring was not routinely available in all cases during the study period and, therefore, was not included as a uniform sedation-monitoring variable in the analysis.

### 2.3. Inclusion and exclusion criteria

#### 2.3.1. Inclusion criteria

Age ≥ 60 years; Scheduled, primary, unilateral hip arthroplasty; American Society of Anesthesiologists (ASA) physical status classification I–III; and Complete medical records.

#### 2.3.2. Exclusion criteria

Emergency or bilateral surgery; Anesthesia method inconsistent or unclear; Use of antiemetic medication within 24 hours preoperatively; preexisting chronic nausea and vomiting-related conditions; History of allergy to related drugs; and Missing key data.

#### 2.3.3. Antiemetic prophylaxis and rescue treatment

During the study period, prophylactic antiemetic administration was not strictly standardized between the 2 anesthesia groups and was determined by the attending anesthesiologist according to patient-related risk factors and institutional practice. The use of any prophylactic or rescue antiemetic agents was extracted from anesthesia records and postoperative nursing records. Patients who received antiemetic medication within 24 hours before surgery were excluded. PONV requiring pharmacological intervention was defined as nausea or vomiting leading to administration of a rescue antiemetic within 24 hours postoperatively. Because antiemetic prophylaxis was not protocolized, this factor was acknowledged as a potential confounder in the Section 4.

### 2.4. Data collection

Data for this study were obtained through retrospective review of hospital electronic medical records and anesthesia information systems. Two researchers, blinded to group allocation, independently extracted the data. Discrepancies were resolved by a third researcher through review of the original records.

#### 2.4.1. Baseline characteristics and surgical information

Collected data included patient age, gender, body mass index (BMI), ASA classification, and comorbidities (hypertension, diabetes, coronary heart disease). Recorded surgical information included: type of surgery (total/hemi hip replacement), duration of surgery, intraoperative blood loss, volume of fluid administration, and preoperative hemoglobin level.

#### 2.4.2. Anesthesia and analgesia information

Group allocation was confirmed based on anesthesia records. For the General Anesthesia Group, the dosages of etomidate, sufentanil, vecuronium, and propofol used for induction and maintenance were recorded. For the Block Group, the dosages of ropivacaine (0.25%, 30 mL) and dexmedetomidine were recorded. The total 24-hour postoperative opioid consumption (converted to morphine milligram equivalents), time to first analgesic request, and rescue analgesia usage were documented for all patients.

#### 2.4.3. Outcome measures information

The primary outcome was the occurrence of PONV within 24 hours postoperatively. The timing (0–6 hours, 6–24 hours), severity, and whether antiemetics were administered were recorded. Secondary outcomes included PACU stay duration, time to first postoperative ambulation, and postoperative hospital length of stay.

#### 2.4.4. Safety indicators information

Adverse events were collected, including intraoperative hypotension, bradycardia, respiratory depression, postoperative delirium, and postoperative urinary retention. For the Block Group, complications such as puncture site hematoma and infection were additionally recorded.

### 2.5. Statistical analysis

All analyses were conducted on the matched sample. Normally distributed continuous data are expressed as mean ± standard deviation and compared using independent samples *t*-tests. Non-normally distributed continuous data are expressed as median (interquartile range) and compared using the Mann–Whitney *U* test. Categorical data are expressed as counts (percentages) and compared using the chi-square test or Fisher exact test. To control for confounding, propensity score matching was performed. Using anesthesia group as the dependent variable, a logistic regression model was constructed with variables including age, sex, BMI, ASA classification, and comorbidities to calculate propensity scores. A 1:1 nearest neighbor matching method was applied with a caliper width of 0.02. Matching effectiveness was assessed by evaluating standardized differences. Multivariate logistic regression analysis was used to identify factors influencing PONV. Variables included in the analysis were anesthesia group, postoperative opioid consumption, age, sex, history of preoperative PONV/motion sickness, and ASA classification. Adjusted odds ratios (aORs) and their 95% confidence intervals (CIs) were calculated. All statistical analyses were performed using Statistical Package for the Social Sciences 26.0 software, with a two-sided *P*-value < .05 considered statistically significant.

Because of the retrospective design, missing data were handled using a complete-case approach. Patients were excluded if key data required for propensity score matching, primary outcome assessment, opioid consumption calculation, or anesthesia classification were missing. No statistical imputation was performed, as the primary outcome and anesthesia-related variables depended on direct medical record documentation.

## 3. Results

### 3.1. Baseline characteristics after propensity score matching

After 1:1 propensity score matching, a total of 100 patients were included for analysis, with 50 patients in each of the Block Group and the General Anesthesia Group. Following matching, there were no statistically significant differences between the 2 groups in baseline characteristics including age, gender, BMI, ASA classification, comorbidities (hypertension, diabetes, coronary heart disease), surgical type, duration of surgery, and preoperative hemoglobin level (all *P* > .05). The absolute values of the standardized mean differences for all variables were <0.20, indicating well-balanced and comparable baseline characteristics between the groups post-matching (Table 1).

**Table 1:** Comparison of baseline characteristics between the 2 groups after propensity score matching (n = 100).

Variable	Block group (n = 50)	General anesthesia group (n = 50)	Statistic	*P*-value	Standardized mean difference (SMD)
Demographics					
Age (yr)	75.3 ± 8.2	73.8 ± 7.5	*t* = 0.971	.334	0.189
Male [n(%)]	23 (46.0)	19 (38.0)	χ^2^ = 0.640	.424	0.161
BMI (kg/m^2^)	24.2 ± 4.1	25.7 ± 3.8	*t* = 1.905	.06	0.178
ASA classification [n(%)]					
Class II	28 (56.0)	31 (62.0)	χ^2^ = 0.373	.541	0.122
Class III	22 (44.0)	19 (38.0)	χ^2^ = 0.373	.541	0.152
Comorbidities [n(%)]					
Hypertension	34 (68.0)	29 (58.0)	χ^2^ = 1.099	.295	0.108
Diabetes	16 (32.0)	12 (24.0)	χ^2^ = 0.800	.371	0.179
Coronary heart disease	9 (18.0)	13 (26.0)	χ^2^ = 0.941	.332	0.194
Surgery-related					
Surgical type [n(%)]					
Total hip arthroplasty	33 (66.0)	29 (58.0)	χ^2^ = 0.667	.414	0.165
Hemiarthroplasty	17 (34.0)	21 (42.0)	χ^2^ = 0.667	.414	0.165
Duration of surgery (min)	108.5 ± 31.2	102.3 ± 28.7	*t* = 1.044	.299	0.106
Preoperative Hb (g/L)	118.7 ± 16.8	122.4 ± 15.3	*t* = 1.166	.247	0.173

ASA = American Society of Anesthesiologists, BMI = body mass index, Hb = haemoglobin.

### 3.2. Comparison of postoperative nausea and vomiting incidence between groups

The overall incidence of PONV within 24 hours postoperatively was significantly lower in the Block Group compared to the General Anesthesia Group (16.0% vs 42.0%, χ^2^ = 8.182, *P* = .004). In subgroup analyses, the incidence of PONV during the 0 to 6 hours postoperative period (10.0% vs 30.0%, *P* = .012) and the incidence of PONV requiring pharmacological intervention (14.0% vs 36.0%, *P* = .012) were both significantly lower in the Block Group. Furthermore, the proportion of patients experiencing moderate-to-severe PONV was higher in the General Anesthesia Group (*P* = .018). There were no statistically significant differences between the 2 groups in the incidence of nausea-only events or events occurring solely in the 6 to 24 hours postoperative period (Table 2).

**Table 2:** Comparison of postoperative nausea and vomiting (PONV) incidence between groups [n(%)].

Variable	Block group (n = 50)	General anesthesia group (n = 50)	χ^2^ value	*P*-value
Primary outcome				
Overall PONV within 24 h	8 (16.0)	21 (42.0)	8.182	.004
Components of PONV				
Nausea only	5 (10.0)	12 (24.0)	3.418	.064
Vomiting (with/without nausea)	3 (6.0)	9 (18.0)	3.374	.066
Severity (worst event)				
No PONV	42 (84.0)	29 (58.0)	8.035	.018
Mild	6 (12.0)	13 (26.0)		
Moderate/Severe	2 (4.0)	8 (16.0)		
Time distribution				
Occurred within 0–6 h Postop	5 (10.0)	15 (30.0)	6.25	.012
Occurred only within 6–24 h Postop	3 (6.0)	6 (12.0)	0.532	.466
PONV requiring pharmacological intervention	7 (14.0)	18 (36.0)	6.25	0.012

### 3.3. Comparison of perioperative medication and surgical data between groups

Regarding anesthesia and analgesic medication, the Block Group demonstrated significantly lower intraoperative sufentanil consumption (18.5 ± 5.2 μg vs 32.8 ± 8.7 μg, *P* < .001) and 24-hour postoperative morphine equivalent dosage (12.3 ± 4.8 mg vs 35.6 ± 9.4 mg, *P* < .001) compared to the General Anesthesia Group. There were no statistically significant differences between the 2 groups in intraoperative blood loss or volume of fluid administration (both *P* > .05) (Table 3).

**Table 3:** Comparison of perioperative medication and surgical data between groups.

Indicator	Block group (n = 50)	General anesthesia group (n = 50)	Statistic	*P*-value
Anesthesia and analgesia medication				
Intraoperative sufentanil dosage (μg)	18.5 ± 5.2	32.8 ± 8.7	*t* = 10.162	<.001
24-h postoperative morphine equivalent dosage (mg)	12.3 ± 4.8	35.6 ± 9.4	*t* = 16.568	<.001
Surgery-related indicators				
Intraoperative blood loss (mL)	185.6 ± 65.4	192.3 ± 70.8	*t* = 0.497	.62
Intraoperative fluid administration (mL)	1250.4 ± 320.7	1315.8 ± 305.2	*t* = 1.048	.297

### 3.4. Comparison of postoperative recovery between groups

In terms of postoperative recovery, the time to first analgesic request was significantly later in the Block Group compared to the General Anesthesia Group (8.5 ± 3.2 hours vs 2.1 ± 1.5 hours, *P* < .001), and the 24-hour rescue analgesia rate was significantly lower (18.0% vs 64.0%, *P* < .001). Both the PACU stay duration (35.2 ± 10.6 min vs 48.7 ± 15.3 min, *P* < .001) and the time to first postoperative ambulation (22.4 ± 6.8 hours vs 28.9 ± 8.5 hours, *P* < .001) were significantly shorter in the Block Group. There was no statistically significant difference in postoperative hospital length of stay between the 2 groups (*P* = .297) (Table 4).

**Table 4:** Comparison of postoperative recovery between groups.

Indicator	Block group (n = 50)	General anesthesia group (n = 50)	Statistic	*P*-value
Analgesia-related indicators				
Time to first analgesic request (h)	8.5 ± 3.2	2.1 ± 1.5	*t* = 13.674	<.001
24-h rescue analgesia rate [n(%)]	9 (18.0)	32 (64.0)	χ^2^ = 21.333	<.001
Recovery process indicators				
PACU stay duration (min)	35.2 ± 10.6	48.7 ± 15.3	*t* = 5.254	<.001
Time to first ambulation (h)	22.4 ± 6.8	28.9 ± 8.5	*t* = 4.334	<.001
Hospitalization duration				
Postoperative hospital stay (d)	7.2 ± 1.8	7.6 ± 2.1	*t* = 1.049	.297

PACU = post-anesthesia care unit.

### 3.5. Logistic regression analysis of factors influencing postoperative nausea and vomiting

Multivariate logistic regression analysis revealed that, after adjusting for postoperative opioid consumption, age, gender, history of preoperative PONV/motion sickness, and ASA classification, the Block Group was an independent protective factor against PONV compared to the General Anesthesia Group (OR = 0.20, 95% CI: 0.08–0.51, *P* = .001). Furthermore, increased postoperative opioid consumption (per 10 mg morphine milligram equivalent, aOR = 1.08, 95% CI: 1.04–1.12, *P* < .001) and a history of preoperative PONV/motion sickness (aOR = 2.44, 95% CI: 1.11–5.37, *P* = .027) were identified as independent risk factors for PONV (Table 5). The absolute risk reduction for 24 hours PONV associated with FICB-sedation was 26.0%, corresponding to a number needed to treat (NNT) of approximately 4. For PONV requiring pharmacological intervention, the absolute risk reduction was 22.0%, with an NNT of approximately 5. For early PONV within 0 to 6 hours, the absolute risk reduction was 20.0%, with an NNT of approximately 5. These estimates suggest that the observed reduction in PONV was not only statistically significant but also clinically meaningful.

**Table 5:** Logistic regression analysis of factors influencing postoperative nausea and vomiting (PONV).

Variable	β value	Standard error	Wald χ^2^	*P*-value	aOR (95% CI)
Anesthesia group (Ref: general anesthesia group)					
Block group	−1.624	0.492	10.857	.001	0.20 (0.08–0.51)
Clinical factors					
Postoperative opioid consumption (per 10 mg MME increase)	0.075	0.019	15.326	<.001	1.08 (1.04–1.12)
Age (per 1 yr increase)	−0.032	0.029	1.224	.269	0.97 (0.92–1.02)
Sex (Ref: female)					
Male	−0.411	0.307	1.789	.181	0.66 (0.36–1.21)
History of preoperative PONV/motion sickness	0.892	0.402	4.921	.027	2.44 (1.11–5.37)
ASA classification (Ref: class II)					
Class III	0.428	0.312	1.884	.17	1.53 (0.83–2.83)

aOR = adjusted odds ratio, ASA = American Society of Anesthesiologists, CI = confidence interval, MME = morphine milligram equivalent, Ref = reference.

### 3.6. Comparison of anesthesia-related adverse events between groups

The safety analysis revealed that the incidence of intraoperative hypotension was significantly lower in the Block Group compared to the General Anesthesia Group (6.0% vs 18.0%, *P* = .042). There were no statistically significant differences between the 2 groups in the incidence of other adverse events, including bradycardia, respiratory depression, postoperative delirium, and postoperative urinary retention (all *P* > .05). In the Block Group, one patient (2.0%) experienced a minor puncture site hematoma, with no occurrences of severe complications such as local anesthetic systemic toxicity (Table 6).

**Table 6:** Comparison of anesthesia-related adverse events between groups [n(%)].

Adverse event	Block group (n = 50)	General anesthesia group (n = 50)	Statistic	*P*-value
Cardiovascular events				
Intraoperative hypotension	3 (6.0)	9 (18.0)	3.296	.042
Bradycardia	2 (4.0)	4 (8.0)	0.182	.67
Respiratory events				
Respiratory depression	1 (2.0)	3 (6.0)	0.206	.65
Neurological events				
Postoperative delirium	3 (6.0)	5 (10.0)	0.133	.715
Other events				
Postoperative urinary retention	4 (8.0)	7 (14.0)	0.696	.404
Puncture site hematoma	1 (2.0)	0 (0.0)	–	1.000*

* Calculated using Fisher exact test.

## 4. Discussion

This retrospective cohort study found that for elderly patients undergoing hip arthroplasty, compared with conventional GA, the anesthesia protocol utilizing ultrasound-guided FICB combined with sedation significantly reduced the incidence of nausea and vomiting within 24 hours postoperatively (16.0% vs 42.0%), while also decreasing intraoperative and postoperative opioid consumption and promoting early patient recovery. These results are highly consistent with the anticipated benefits of the opioid-sparing multimodal analgesia strategy centered on regional blocks, which has been advocated in recent years.^[10]^ The core mechanism is likely that the FICB provides continuous and stable analgesia for the surgical area, substantially reducing the need for systemic opioid analgesics at the source. It is well established that opioids, by activating μ-receptors in the chemoreceptor trigger zone of the medulla, are the primary and dose-dependent pharmacological factor triggering PONV.^[11]^ The multifactorial regression analysis in this study provides direct evidence for this: for every 10 mg increase in morphine equivalent postoperative opioid consumption, the risk of PONV increased by 8% (aOR = 1.08), while being in the block group itself emerged as an independent protective factor against PONV (aOR = 0.20). This strongly suggests that the PONV benefit conferred by regional blockade is largely mediated through the pathway of the “opioid-sparing effect.”^[12]^

It is noteworthy that the reduction in PONV incidence was not only reflected in the overall numbers but also had significant clinical detail. The Block Group demonstrated significantly lower rates of both early postoperative (0–6 hours) PONV and PONV requiring pharmacological intervention, with fewer cases of moderate-to-severe PONV. This aligns with the period of emergence from anesthesia and the immediate postoperative phase, which are characterized by the strongest residual effects of opioids and pain stimuli. The regional block provided seamless analgesia during this critical period, avoiding analgesic gaps and additional opioid load.^[13]^

Furthermore, the Block Group demonstrated comprehensive advantages in recovery quality: a significantly delayed time to first analgesic request, a reduced need for rescue analgesia, and shorter durations for both PACU stay and time to first ambulation. These findings collectively depict a smoother postoperative recovery trajectory. Early ambulation is a cornerstone for preventing complications and achieving functional recovery after hip arthroplasty.^[14]^ Regional blockade creates favorable conditions for achieving this goal by providing superior analgesia while reducing sedation- and opioid-related side effects (such as dizziness and drowsiness).^[15]^

Regarding safety, the Block Group exhibited better hemodynamic stability, with a significantly lower incidence of intraoperative hypotension compared to the General Anesthesia Group. This is likely attributable to the regional block avoiding the depression of vascular tone and myocardial contractility caused by general anesthetic agents (such as propofol, volatile anesthetics) and high-dose opioids.^[16]^ Maintaining intraoperative circulatory stability holds significant clinical importance for the elderly population, who have a high prevalence of cardiovascular comorbidities. Concurrently, the incidence of severe block-related complications (such as local anesthetic systemic toxicity, nerve injury) was zero in this study, with only one case of minor hematoma. This further supports that ultrasound-guided FICB is a procedure with a high safety profile.^[17]^

Of course, this study also has several limitations. Firstly, the retrospective design itself cannot completely avoid selection bias and confounding. Although we employed propensity score matching, a rigorous statistical method to simulate randomization and balance observable baseline variables, there may still be unmeasured potential confounding factors (such as surgical skill, variations in rehabilitation protocols, etc) influencing the outcomes.^[18]^ Secondly, a potential limitation of this study is that prophylactic antiemetic administration was not fully standardized between the 2 groups. Although patients who received antiemetic medication within 24 hours before surgery were excluded and rescue antiemetic use was captured as an outcome-related variable, differences in intraoperative prophylactic antiemetic practice may have influenced the observed incidence of PONV. This is particularly relevant because current PONV guidelines recommend risk-based assessment and multimodal prophylaxis, especially in patients with risk factors. Therefore, the lack of a standardized antiemetic protocol should be considered a residual confounder, and future prospective studies should apply uniform antiemetic prophylaxis to better isolate the effect of the anesthesia technique itself.^[19]^ Thirdly, regarding the effectiveness of the regional block, we relied on medical record documentation and were unable to precisely assess the actual sensory block extent for each patient. The inclusion of a few cases with incomplete block might have occurred, potentially underestimating the true advantage of the regional technique. Fourth, this was a single-center study conducted in an elderly Chinese population, which may limit external validity. Patient characteristics, body habitus, baseline PONV risk, anesthesia practice, ERAS implementation, rehabilitation protocols, and the availability and expertise of ultrasound-guided regional anesthesia may differ across institutions and countries. Therefore, the findings should be generalized cautiously to other healthcare systems, ethnic populations, and perioperative pathways. Multicenter prospective studies are needed to determine whether similar benefits can be reproduced in broader clinical settings. Finally, this study primarily focused on short-term in-hospital outcomes and lacks follow-up data on long-term patient pain, functional recovery, and the incidence of chronic pain.^[20]^ The clinical feasibility of the FICB-sedation strategy should also be considered. This approach may be particularly attractive for elderly patients at high risk for PONV, opioid intolerance, or hemodynamic instability, because it can reduce systemic opioid exposure and avoid some physiological effects of conventional GA. However, successful implementation requires experienced ultrasound-guided regional anesthesia providers, careful sedation titration, continuous respiratory and hemodynamic monitoring, and a clear plan for conversion to GA if analgesia or sedation is inadequate. Patients with severe cognitive impairment, inability to cooperate, severe anxiety, local infection, coagulopathy, allergy to local anesthetics, or anticipated difficulty maintaining a safe sedation state may not be ideal candidates. Thus, appropriate patient selection remains essential.

## 5. Conclusion

In this retrospective propensity score–matched cohort study of elderly patients undergoing hip arthroplasty, ultrasound-guided FICB combined with sedation was associated with a lower incidence of 24 hours PONV, reduced intervention-requiring and early PONV, lower opioid consumption, shorter PACU stay, earlier ambulation, and fewer episodes of intraoperative hypotension compared with conventional GA. These findings support the potential value of an opioid-sparing block-sedation strategy within ERAS-oriented perioperative care. However, because of the retrospective single-center design and the lack of a standardized antiemetic protocol, the results should be interpreted cautiously and confirmed in future multicenter randomized controlled trials.

## Author contributions

**Conceptualization:** Qingtang Yin, Daobing Chen, Chunmiao Gu, Beibei Zhang.

**Data curation:** Qingtang Yin, Daobing Chen, Chunmiao Gu, Beibei Zhang.

**Formal analysis:** Qingtang Yin, Daobing Chen, Chunmiao Gu, Beibei Zhang.

**Funding acquisition:** Qingtang Yin, Daobing Chen, Chunmiao Gu, Beibei Zhang.

**Investigation:** Qingtang Yin, Beibei Zhang.

**Writing – original draft:** Beibei Zhang.

**Writing – review & editing:** Beibei Zhang.
